# Effects of luteinizing hormone-releasing hormone and arginine-vasotocin on the sperm-release response of Günther's Toadlet, *Pseudophryne guentheri*

**DOI:** 10.1186/1477-7827-8-139

**Published:** 2010-11-08

**Authors:** Aimee J Silla

**Affiliations:** 1 School of Animal Biology, The University of Western Australia, Perth, Australia

## Abstract

**Background:**

Luteinizing hormone-releasing hormone (LHRH) is an exogenous hormone commonly used to induce spermiation in anuran amphibians. Over the past few decades, the LHRH dose administered to individuals and the frequency of injection has been highly variable. The sperm-release responses reported have been correspondingly diverse, highlighting a need to quantify dose-response relationships on a species-specific basis. This study on the Australian anuran *Pseudophryne guentheri *first evaluated the spermiation response of males administered one of five LHRHa doses, and second, determined whether AVT administered in combination with the optimal LHRHa dose improved sperm-release.

**Methods:**

Male toadlets were administered a single dose of 0, 1, 2, 4 or 8 micrograms/g body weight of LHRHa. A 4 micrograms/g dose of AVT was administered alone or in combination with 2 micrograms/g LHRHa. Spermiation responses were evaluated at 3, 7 and 12 h post hormone administration (PA), and sperm number and viability were quantified using fluorescent microscopy.

**Results:**

LHRHa administration was highly effective at inducing spermiation in *P. guentheri*, with 100% of hormone-treated males producing sperm during the experimental period. The number of sperm released in response to 2 micrograms/g LHRHa was greater than all other doses administered and sperm viability was highest in the 1 microgram/g treatment. The administration of AVT alone or in combination with LHRHa resulted in the release of significantly lower sperm numbers.

**Conclusion:**

Overall, results from this study suggest that in *P. guentheri*, LHRHa is effective at inducing spermiation, but that AVT inhibits sperm-release.

## Background

Amphibian populations are declining worldwide at a rate unparalleled by any other vertebrate group [1]. In an attempt to impede this unprecedented loss of amphibians, captive assurance colonies are being established for threatened and endangered species. Unfortunately breeding failures are common due to the inherent difficulties associated with simulating the specific environmental conditions required to trigger anurans to breed in captivity [2]. As a result, captive breeding facilities are now focusing their attention to assisted reproductive technologies (ART) [2].

An integral component of assisted reproduction is the reliable and effective collection of spermatozoa for cryopreservation and artificial fertilisation (AF). Traditionally, collection of anuran spermatozoa has been facilitated by the removal and maceration of testes from euthanized individuals [3]. This method has been widely used because of the ease and reliability at which high concentrations of spermatozoa can be obtained, but research in reproductive biology is now favouring the use of non-invasive techniques that can be applied to live individuals [4]. Exogenous hormones have been used to induce spermiation (sperm release) in a number of anuran [5-11] and urodele [10,12,13] species since the early 20^th ^century. One of the hormones more commonly used to induce spermiation is luteinizing hormone-releasing hormone (LHRH). LHRH is a tropic peptide hormone that stimulates the anterior pituitary gland to release luteinizing hormone (LH). If a sufficient dose of LHRH is administered it will trigger the acute rise in LH necessary to stimulate spermiation. Although LHRH has been used to induce spermiation over the past few decades the dose applied to individuals, as well as the method and frequency of LHRH administration, has been highly variable [14]. The resultant spermiation responses have been correspondingly diverse, highlighting a need to quantify dose-response relationships.

Amphibian species appear to vary considerably in their sensitivity to LHRH administration, despite the conservation of the structure and function of LHRH among vertebrates. Goncharov et al. [10] induced 39 amphibian species to spawn following administration of a synthetic LHRH analogue, but the concentration of LHRHa required was highly variable. In some species, individuals could be induced to spawn following a single injection of 0.0002 μg/g, while others required repeated injections in excess of 0.8 μg/g, representing a 4000-fold range in LHRHa potency. In response to the observed variation in spermiation responses, Kouba et al. [2] recently recognised an urgent need to develop dose-response curves on a species-specific basis as opposed to the trial-and-error approaches commonly used by zoological institutions.

If doses of LHRH administered are too low for a given species, they will induce LHRH-receptor synthesis (up-regulation) without a change in serum or pituitary LH content [15,16]. As the LHRH dose administered increases, nearing the optimal concentration, up-regulation of LHRH receptors continues, receptor numbers are elevated and the tissue responds with an LH surge [16] inducing an increasingly positive spermiation response. Where LHRH doses administered are above the optimal concentration for a species, pituitary desensitization and/or down-regulation of LHRH-receptors occurs, reducing LH release and impeding the spermiation response [15]. Administration of extreme supraphysiological doses further leads to the down-regulation of LHRH-receptors at both the pituitary and gonadal levels, blocking testis activation and inducing temporary antifertility effects [15].

In addition to a need to identify optimal LHRH doses, there is a growing interest in trialling the use of combinations of hormones to improve the efficacy of gamete-release induction protocols [17,18]. The neurohypophysial peptide arginine vasotocin (AVT) has long been recognised for its role in the reproductive behaviours of amphibians such as male and female vocalization [19,20], pheromone release [21] and amplectic clasping [22,23]. Additionally AVT has been shown to induce smooth muscle contractions of the oviduct [24,25] and has subsequently been used to induce partuition and oviposition in a number of amphibian and reptile species [23,26]. Zoeller et al [27] were the first to propose a link between AVT and the transport of spermatozoa into the cloaca in amphibians, having successfully elicited contraction of the Wolffian ducts in the rough-skinned newt (*Taricha granulosa) in vitro*. In support of these findings, a study of the red-bellied newt (*Cynops pyrrhogaster*) quantified a dose-dependent induction of spermatophore deposition to administration of AVT *in vivo *[21]. Based on these results, it is hypothesised that AVT will enhance the timing and/or the number of spermatozoa released in anuran amphibians.

To address the lack of dose-response data and to investigate the possible synergy of simultaneous administration of LHRHa and AVT, this study used the Western Australian anuran *Pseudophryne guentheri*, to: 1) evaluate the dose-response relationship of changes in sperm count, sperm viability and timing of sperm release in response to LHRHa administration; and 2) determine whether AVT administered in combination with the optimal LHRHa dose alters the sperm-release response.

## Methods

The procedures described in this manuscript were conducted following evaluation and approval by the University of Western Australia's Animal Ethics Committee (approval number RA/3/100/869).

### Study species

*Pseudophryne guentheri *is a small (26-33 mm, snout-vent length) stout-bodied toadlet (family: Myobatrachidae) endemic to south Western Australia. The breeding season of this species is prolonged, commencing in autumn following heavy rainfall and continuing for several weeks. Male *P. guentheri *aggregate in low lying areas that are subject to seasonal inundation where they construct and defend terrestrial burrows. To attract mates, male *P. guentheri *produce advertisement calls that are broadcast from the entrance of the burrow. Following successful courtship, which takes place below the soil surface, females deposit a small clutch of eggs (range = 80-410, mean = 224 ± 12, n = 40, Silla unpublished data). The developing embryos remain encapsulated until the burrow floods in winter triggering the tadpoles to hatch into temporary pools where they complete development. This reproductive mode (terrestrial embryonic development, aquatic larvae) is consistent with the pattern of development exhibited by other species in the genus *Pseudophryne *[28].

### Study site and animal collection

Male *P. guentheri *were collected from a breeding chorus located in the Pinjar Wetlands, approximately 40 km north of Perth, Western Australia. All collections took place between 18:00 and 24:00 hours from June 1-19 2009. Terrestrial nests were located by triangulating male vocalisations and the resident toadlets were collected by hand. The toadlets were identified as mature males from their calling behaviour and the presence of pigmented vocal sacs. Within four hours of collection, males were transported to a constant temperature room set to a 17°C day/12°C night temperature cycle and maintained under artificial illumination (10.5 h light/13.5 h dark). Animals were housed individually in plastic aquaria (220 mm L × 140 mm W × 160 mm H) containing a layer of moist sponge beneath 10-12 cm of soil, which allowed males to burrow for shelter. Toadlets were randomly assigned to one of seven treatment groups (see 'hormone administration' section below) and there were no significant differences (ANOVA: F_6, 63 _= 1.49, *p*= 0.20) in the size of animals between treatments (n = 64, average mass = 2.79 ± 0.04 SEM). An 'acclimation period' of 4-6 days was allowed before experiments commenced in order to minimise potential effects of collection stress, and elevated corticosterone levels, on the efficacy of hormone treatment.

### Hormone administration

This experiment was conducted in two stages; the first involved establishing a spermiation dose-response relationship to four doses of LHRHa (see 'stage one' below) and the second stage tested the ability of AVT (see 'stage two' below), administered in combination with the optimal dose of LHRHa (as identified in stage one), to improve sperm release. The experiment was conducted from 6/6/09 to 1/7/09 with an interval of 6 days between stage one and two.

*Stage one: Effect of LHRHa dose on spermiation response- *The spermiation response of male *P. guentheri *administered a single dose of 1 μg, 2 μg, 4 μg or 8 μg/g body weight LHRHa (Leuprorelin acetate; Lucrin^®^) was quantified. Hormones were diluted in 100 μL of Simplified Amphibian Ringer (113 mM NaCl, 2 mM KCl, 1.35 mM CaCl_2_, 1.2 mM NaHCO_3_) and administered to males via subcutaneous injection into the dorsal lymph sac. A control treatment consisted of toadlets administered 100 μL of Simplified Amphibian Ringer (SAR), the vehicle for hormone administration. The experimental treatments and the control treatment all involved a sample size of 10 toadlets. Immediately prior to hormone application, *P. guentheri *were weighed to the nearest 0.01 g and the dose administered was adjusted according to an individual's body mass.

*Stage two: Effect of LHRHa and AVT on spermiation response- *To determine the effect of administering LHRHa (Leuprorelin acetate; Lucrin^®^) in combination with AVT (arg^8^- vasotocin acetate salt; Sigma-Aldrich) on the sperm-release response of *P. guentheri *the optimal LHRHa dose (as identified by stage one above) was administered in combination with 4 μg/g AVT (n = 7). An additional 7 males received 4 μg/g AVT in the absence of LHRHa as a positive control. Hormones were diluted in 100 μL of SAR and injected into the toadlet's dorsal lymph sac as described above.

Following hormone administration all individuals were returned to separate plastic holding tanks (50 mm D × 90 mm H) containing three pieces of moist sponge (20 mm W × 20 mm L × 3 mm H). Adopting this procedure ensured sufficient hydration of the males to permit the collection of urine throughout the sampling period. To minimise faecal contamination of the urine samples, toadlets were not fed during the four days prior to experimentation.

### Collection and assessment of spermic urine

Spermic urine samples were collected at 3, 7 & 12 h (± 10 mins) after hormone administration. The collection method involved placing the end of a glass microcapillary tube (fire polished and cooled) into the cloaca and massaging the walls of the cloacal opening with the tube until urination occurred. Once collected, spermic urine volume was measured by placing the microcapillary tube alongside a ruler and the sample was then prepared for assessment of sperm yield and sperm viability using fluorescent microscopy. Spermic urine was homogenized with 5 μL of a 1:50 dilution of the nucleic acid stain SYBR-14 (Invitrogen L-7011) and incubated in the dark for 7 min. A 2 μL aliquot of Propidium iodide was added and the solution was incubated in the dark for a further 7 min. A wet mount was prepared and sperm viability evaluated by fluorescent microscopy under x20 magnification at a wavelength of 490 nm. Spermatozoa fluorescing bright green were considered viable, while those exhibiting red fluorescence were considered non-viable [29]. The total sperm count and proportion of viable sperm per sample was quantified for the absolute volume of urine collected for each sample.

### Statistical analyses

The number of spermiating toadlets was compared between LHRHa dose treatments (1 μg/g, 2 μg/g, 4 μg/g or 8 μg/g), and between each dose treatment and the control, using two-tailed Fisher exact tests. The number of spermatozoa expelled and sperm viability were compared between treatments using one-way analysis of variance (ANOVA) and Tukey-Kramer Honestly Significant Difference (HSD) *post hoc *tests. Bartlett's tests were conducted on all variables to determine homogeneity of variances prior to all other analyses. Data on the number of spermatozoa expelled were log transformed, using the transformation log_10_(x+1) and sperm viability data was arcsine transformed using the transformation sin^-1^(√x). Comparison of the number of spermatozoa expelled at 7 h and 12 h were analysed using Welch's ANOVAs due to unequal variances after transformation. All statistical analyses were performed using the JMP 8.0.1 software package (SAS Institute Inc. 2009). For all tests in this study, *P < 0.05 *was considered significant.

## Results

### Effect of LHRHa dose on spermiation response

**Figure 1:**
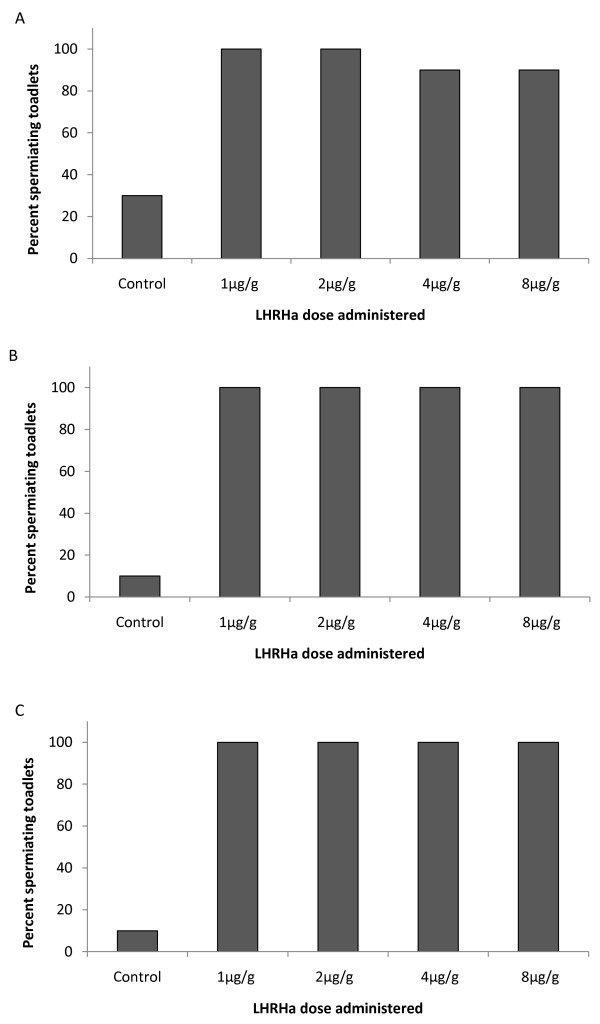
**Number of spermiating toadlets**. The percentage of males releasing sperm at A) 3 h, B) 7 h and C) 12 h PA.

Urine samples were successfully collected from all individuals at each sampling period (3, 7 & 12 h) post administration (PA), with urine volumes ranging from 2 -152 μL (mean = 21.29 ± 1.59 μL). Of the urine samples obtained from males administered 1 μg/g, 2 μg/g, 4 μg/g or 8 μg/g LHRHa, 90-100% contained spermatozoa within 3 h PA (figure 1A). The number of spermiating males further increased at subsequent sampling periods, such that 100% of individuals in each of the four dose treatments (1 μg/g, 2 μg/g, 4 μg/g or 8 μg/g) were responsive at 7 h and 12 h PA (figure 1 B &1C). The majority of samples obtained from control animals were aspermic, however 10-30% of individuals within this treatment released spermatozoa at each sampling period (figure 1). The number of spermiating males was significantly higher in hormone treatments relative to the control treatment, but the number of spermiating males administered varying doses of LHRHa (1 μg/g, 2 μg/g, 4 μg/g or 8 μg/g) did not differ significantly from one another (Tab. 1).

**Table 1:** Comparison of the number of spermiating males administered LHRHa (*n *= spermiating toadlets/toadlets within treatment group)

	Control (*n *= 4/10)	1 μg/g (*n *= 10/10)	2 μg/g (*n *= 10/10)	4 μg/g (*n *= 10/10)	8 μg/g (*n *= 10/10)
**Control **(*n *= 4/10)		0.011*	0.011*	0.011*	0.011*
**1 μg/g **(*n *= 10/10)	0.011*		1.000	1.000	1.000
**2 μg/g **(*n *= 10/10)	0.011*	1.000		1.000	1.000
**4 μg/g **(*n *= 10/10)	0.011*	1.000	1.000		1.000
**8 μg/g **(*n = *10/10)	0.011*	1.000	1.000	1.000	

Data shown are *P *values generated from two-tailed Fisher Exact Tests. * denotes statistical significance (P < 0.05).

**Figure 2:**
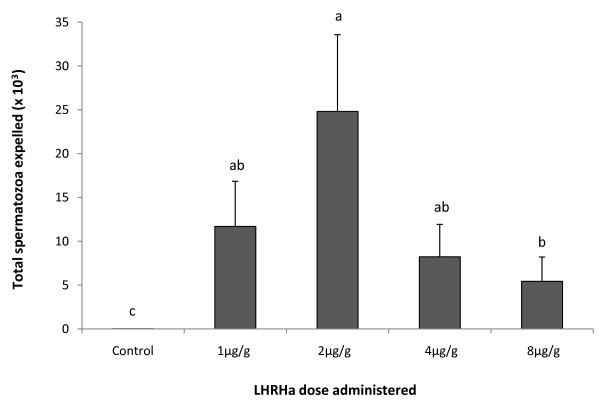
**Total number of spermatozoa (× 10^3^) released by males administered LHRHa (n = 10) over a 12 h sampling period**. Data shown are untransformed mean ± SEM. Letters displayed are the result of a Tukey Kramer HSD post-hoc test of Log_10 _(x +1) transformed data. Treatments that share a letter are not significantly different from each other.

The total number of spermatozoa expelled over a 12 h period PA differed significantly according to dose treatment (one-way ANOVA, F_4,49 _= 43.16, *p *< 0.001; figure 2). The 2 μg/g treatment produced a significantly higher number of spermatozoa compared to the control and 8 μg/g treatments (Tukey-Kramer HSD, *P *< 0.05; figure 2), but was not significantly higher than to the 1 μg/g or 4 μg/g treatments (Tukey-Kramer HSD, *P *> 0.05; figure 2). In addition to the treatment effect identified for the total spermatozoa expelled, significant treatment effects were also detected at each of the individual sampling times, 3 h (one-way ANOVA, F_4,49 _= 13.22, *p *< 0.001), 7 h (Welch's ANOVA, F_4 _= 132.32, *p *< 0.001) and 12 h (Welch's ANOVA, F_4 _= 74.64, *p *< 0.001) PA. The number of spermatozoa expelled by males in the 2 μg/g treatment was consistently higher than the remaining treatments at all sampling periods PA (Tab. 2). Peak sperm-release occurred at 7 h PA for males administered 8 μg/g LHRHa, while all remaining dose treatments produced the highest number of spermatozoa at 12 h PA (Tab. 2). The total number of spermatozoa expelled was not related to the volume of urine collected or to toadlet mass (r^2 ^= 0.003, *p *= 0.724; r^2 ^= 0.008, *p *= 0.549, respectively).

**Table 2:** The number of spermatozoa released (× 10^3^) and sperm viability of samples collected at 3, 7 and 12 h post LHRHa administration

	3 h PA		7 h PA		12 h PA	
**Treatment**	**Sperm Count (× 10^3^)**	**Sperm Viability**	**Sperm Count (× 10^3^)**	**Sperm Viability**	**Sperm Count (× 10^3^)**	**Sperm Viability**
Control	0.002 ± 0.002	-	0.0002 ± 0.0002	-	0.0009 ± 0.0009	-
1 μg/g	2 .79 ± 1 .57	0.62 ± 0.09	3 .36 ± 1 .55	0.67 ± 0.05	15 .54 ± 2 .41	0.71 ± 0.07
2 μg/g	6 .07 ± 2 .06	0.56 ± 0.05	5 .04 ± 2 .27	0.48 ± 0.08	13 .69 ± 6 .27	0.54 ± 0.06
4 μg/g	0 .66 ± 0 .33	0.66 ± 0.10	3 .20 ± 1 .47	0.62 ± 0.06	14 .29 ± 2 .38	0.57 ± 0.08
8 μg/g	0 .87 ± 0 .64	0.64 ± 0.09	2 .63 ± 2 .05	0.43 ± 0.08	0 .51 ± 0 .21	0.41 ± 0.08

Data shown are means ± SEM.

The proportion of viable sperm (sperm viability) was calculated for all samples where a sperm count of ≥30 spermatozoa was achieved. The overall mean sperm viability differed significantly according to dose treatment (one-way ANOVA, F_3,38 _= 3.07, *p *= 0.040), with toadlets injected with 1 μg/g producing spermic urine samples of significantly higher sperm viability than those administered 8 μg/g LHRHa (0.667 ± 0.045 vs 0.489 ± 0.036; Tukey-Kramer HSD, *P *< 0.05). Males within the 2 μg/g and 4 μg/g treatments produced spermic urine samples of intermediate sperm viability which were not significantly different from any of the remaining treatments (0.524 ± 0.047; 0.605 ± 0.054, respectively; Tukey-Kramer HSD, *P *> 0.05). The temporal effect of sampling period (3, 7 & 12 h) on sperm viability did not affect the dose treatments consistently. The sperm viability of samples collected from toadlets in the 4ug/g and 8ug/g treatments decreased over time, while the sperm viability of spermic urine samples collected from males in the 1ug/g treatment increased with increasing time PA (Tab. 3). The sperm viability of samples in the 2ug/g treatment decreased slightly at 7 h PA before increasing again at 12 h PA (Tab. 2).

### Effect of LHRHa and AVT on spermiation response

**Figure 3:**
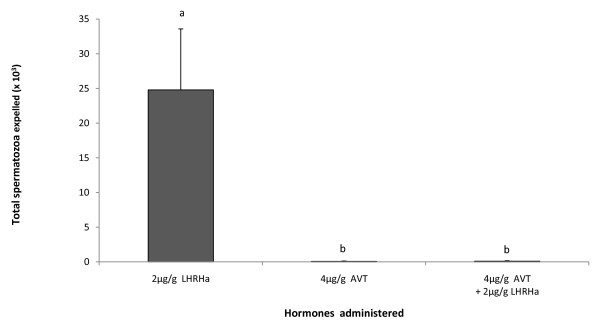
**Total number of spermatozoa (× 10^3^) released by males administered LHRHa (n = 10), AVT (n = 7) and AVT + LHRHa (n = 7) over a 12 h sampling period**. Data shown are untransformed mean ± SEM. Letters displayed are the result of a Tukey Kramer HSD post-hoc test of Log_10 _(× +1) transformed data. Treatments that share a letter are not significantly different from each other.

The number of male toadlets spermiating in response to the administration of 2ug/g LHRHa (10/10 males) was not significantly different to the number of spermiating males administered either 4ug/g AVT (5/7 males) or the combination of 2ug/g LHRHa +4ug/g AVT (5/7 males)(two-tailed Fisher Exact Tests, *p *> 0.05). In contrast, the difference in the number of spermatozoa expelled from males across these three treatments was highly significant (one-way ANOVA, F_2,23 _= 38.865, *p *< 0.001). The 2 μg/g treatment produced significantly greater sperm counts than the AVT and AVT + LHRHa treatments (Tukey-Kramer HSD, *P *< 0.05; figure 3), which both yielded mean sperm counts of less than 100 spermatozoa. The viability of spermatozoa collected from toadlets administered LHRHa, AVT and LHRHa+AVT (0.524 ± 0.047; 0.453 ± 0.216; 0.560 ± 0.200, respectively) did not differ significantly (one-way ANOVA, F_2,14 _= 0.144, *p *= 0.868).

## Discussion

To date, studies investigating the hormonal induction of spermiation in amphibians *in vivo *have largely failed to quantify dose-response relationships, despite the high variation of hormone potency observed between species. This study quantified the sperm count, sperm viability and timing of sperm release of *P. guentheri *in response to five doses of LHRHa, and also determined whether AVT administered in combination with the optimal dose of LHRHa improved the sperm-release response.

### Effect of LHRHa dose on spermiation response

The results of this study showed that administration of LHRHa at doses between 1 μg- 8 μg/g body weight were highly successful at inducing *P. guentheri *to spermiate. Between 90 and 100% of males released spermatozoa within 3 h of treatment, and 100% of males released spermatozoa at 7 and 12 h PA. The number of spermiating males did not differ according to dose administered, but as expected, the number of spermatozoa expelled between dose treatments varied significantly. Peak sperm release occurred in the 2 μg/g treatment, with a large drop in the number of spermatozoa expelled in the 4 μg/g treatment, and a further decline identified in the 8 μg/g treatment. This reduction in the number of spermatozoa expelled in the higher dose treatments could be a result of LHRH-receptor down-regulation, where LHRH doses above optimal levels induce a decline in the number of LHRH-receptors, reducing LH release, and subsequently impeding the spermiation response [15]. Results from this experiment indicate that studies attempting to induce spermiation in *P. guentheri *might avoid pituitary desensitization and/or LHRH-receptor down regulation by administering LHRH at doses below 4 μg/g.

Concentrations of synthetic LHRHa administered to induce spermiation in anurans are highly variable, but typically do not exceed 0.8 μg/g per injection [7,10,30,31]. For example, in the South American frog, *Lepidobatrachus laevis *a dose range of 0.288-0.579 μg/g induced the release of an average of 6.2 × 10^6 ^spermatozoa per male [30]. Similarly, a recent study in the Australian hylid *Litoria peronii *successfully induced spermiation by administering a dose of 0.75 μg/g LHRHa [31]. The optimal dose identified in this study was substantially higher (2 μg/g). This result may be a consequence of *P. guentheri *being less sensitive to LHRHa than the majority of previous anuran species studied. Alternatively, given the current lack of empirical dose-response studies in anurans, coupled with high spermiation failure rates for many species, it may be that the LHRHa doses typically used to induce spermiation in anurans are sub-optimal.

With respect to sperm viability, analysis of spermic urine samples revealed that the 1 μg/g treatment produced a higher proportion of live sperm than the 8 μg/g treatment, suggesting that lower LHRHa doses induce the release of higher quality spermatozoa in this species. Overall, average sperm viability ranged from 48.9% to 66.7% between dose treatments. This range is considerably lower than the > 84% reported by Rowson et al. [14] for *Bufo americanus *and *B. valliceps *and the 97.5% and 95.3% reported by Obringer et al. [7] for *B. americanus *and *B. baxter*. The highest sperm viability reported in the present study was also at the lower end of the range of 58% to 97% reported by for the Australian hylid *Litoria peronii *[31].

Sperm viability is an important indicator of the fertilizing capacity of spermatozoa. Obtaining spermatozoa of maximum viability is therefore an important step toward successful artificial fertilizations, and should be the focus of future ART research in *P. guentheri*. The comparatively low sperm viability reported in this study may be due to two factors. First, it may be an artefact of the sampling intervals employed and the latency period during which spermatozoa remained in the cloaca after spermiation. Spermatozoa released within minutes of the sampling period may remain viable, whereas those spermatozoa released hours earlier may not survive a prolonged period in the cloaca. This would be reflected by a moderate to poor sperm viability overall, and may be avoided by reducing the sampling period to hourly or half-hourly intervals. Alternatively, reduced sperm viability may be a reflection of incomplete spermatogenesis and capacitation of spermatozoa prior to spermiation. This may be elucidated in future studies by examining histological sections of the testes.

Incomplete gamete maturation may be improved by altering hormone administration protocols. For example, aquaculture research has long acknowledged the beneficial effect of multiple hormone injections and controlled-release delivery systems on broodstock spawning (for a review see [32]). These methods of pulsatile hormone administration may work to 'prime' the hypothalamic-pituitary-gonadal axis, promoting gametogenesis and final gamete maturation prior to gamete release. In the Wyoming Toad, *Bufo baxteri *multiple low-dose priming injections administered prior to a higher ovulatory dose have been successful in promoting the ovulation of more oocytes of better quality [8]. Low dose priming protocols may similarly improve spermiation in male anurans, but are yet to be empirically tested.

Another method used to improve gamete release in lower vertebrates is the administration of dopamine antagonists (e.g., domperidone, pimozide, reserpine or metoclopramide) prior to, or in combination with, the administration of LHRHa [17,18,32,33]. Dopamine inhibits the release of gonadotropins from the pituitary, reducing the stimulatory effect of LHRHa on LH release [32]. Dopamine antagonists are therefore used to prevent the release of dopamine or inhibit its binding in order to enhance the LHRHa-induced release of viable gametes [33]. Recently, the combined administration of LHRHa with metoclopramide was shown to be effective in increasing spawning rates of four American frog species in captivity [18]. The use of dopamine antagonists may therefore be a useful tool in improving the spermiation response of *P. guentheri *in future ART research.

### Effect of LHRHa and AVT on spermiation response

The response of *P. guentheri *to the administration of LHRHa, AVT, and LHRHa in combination with AVT, clearly showed that spermiation is most effectively induced by LHRHa alone. The administration of AVT has been shown to exert a positive effect on sperm deposition in newts [21,23]. However, contrary to the study by Moore et al. [23], the concomitant administration of AVT with LHRHa in *P. guentheri *did not improve the spermiation response, and instead had an inhibitory effect. The inhibition of gonadal activity by AVT has also been reported in other vertebrate taxa, including reptiles and mammals [34-37]. Additionally, Yamashita et al. [38] discovered that the mechanism underlying the antigonadal action of AVT in the dog involved the AVT-induced suppression of LH release by the anterior pituitary. Their study showed that AVT administered prior to LHRH inhibited testicular activity, but that AVT did not affect testicular activity when administered prior to human chorionic gonadotropin (hCG). The reproductive hormone hCG acts by mimicking LH and therefore has the ability to bypass the inhibitory effect of AVT on LH secretion and acts directly on the gonads. The inhibitory effect of AVT on spermiation reported in this study may be a result of the use of AVT in combination with LHRHa. Future anuran spermiation studies may benefit from administering AVT in combination with hCG instead of LHRHa, or by delaying the administration of AVT so that LHRHa can first induce its effect on the gonads.

## Conclusion

This study showed that LHRHa is highly effective at inducing spermiation in the Günther's Toadlet, *P. guentheri*. The maximal number of spermatozoa were expelled in response to the administration of 2 μg/g LHRHa, above all other doses trialled (0, 1, 4, or 8 μg/g). The concomitant administration of LHRHa with AVT did not improve the spermiation response and instead inhibited the expulsion of spermatozoa. The hormone protocols used induced the release of spermatozoa of lower sperm viability than those reported in studies of other anuran species. The comparatively low sperm viability reported in this study may be a consequence of the collection latency period during which spermatozoa remained in the cloaca, or may indicate there was incomplete spermatogenesis prior to spermiation. The refinement of current spermiation protocols to improve sperm viability should be prioritized in future research developing artificial reproduction in *P. guentheri*.

## Competing interests

The author declares that there are no competing interests.

## Authors' contributions

AJS designed the study, collected and housed the study animals, performed all experimental procedures and wrote the paper.

## References

[B1] Stuart SN Chanson JS Cox NA Young BE Rodrigues ASL Fischman DL Waller RW Status and trends of amphibian declines and extinctions worldwide Science 2004 306 1783 1786 10.1126/science.1103538 15486254

[B2] Kouba AJ Vance CK Willis EL Artificial fertilization for amphibian conservation: Current knowledge and future considerations Theriogenology 2009 71 214 227 10.1016/j.theriogenology.2008.09.055 19026442

[B3] Rugh R Experimental embryology: a manual of techniques and proceedures 1948 Minneapolis, Minnesota: Burgess Publishing Company

[B4] Kouba AJ Vance CK Applied Reproductive technologies and genetic resource banking for amphibian conservation Reprod Fertil Dev 2009 21 719 737 10.1071/RD09038 19567216

[B5] Mann RM Hyne RV Choung CB Hormonal induction of spermiation, courting behaviour and spawning in the Southern Bell Frog, Litoria raniformis Zoo Biol 2010 29 1 9 20549714 10.1002/zoo.20331

[B6] Creaser CW Gorbman A Species specificity of the gonadotrophic factors in invertebrates Q Rev Biol 1939 14 311 331 10.1086/394589

[B7] Obringer AR O'Brien JK Saunders RL Yamamoto K Kikuyama S Roth TL Characterization of the spermiation response, luteinizing hormone release and sperm quality in the American toad (*Bufo americanus*) and the endangered Wyoming toad (*Bufo baxteri*) Reprod Fertil Dev 2000 12 51 58 10.1071/RD00056 11194557

[B8] Browne RK Seratt J Vance C Kouba A Hormonal priming, induction of ovulation and in-vitro fertilization of the endangered Wyoming toad (*Bufo baxteri*) Reprod Biol Endocrinol 2006 4 10.1186/1477-7827-4-34 PMC1524778 16790071

[B9] Minucci S Di Matteo L Baccari GC Pierantoni R A gonadotropin releasing hormone analog induces spermiation in intact and hypophysectomized frogs, *Rana esculenta* Experientia 1989 45 1118 1121 10.1007/BF01950175 2513222

[B10] Goncharov BF Shubravy OI Serbinova IA Uteshev VK The USSR programme for breeding amphibians, including rare and endangered species Int Zoo Yearbk 1989 28

[B11] Pozzi AG Rosemblit C Ceballos NR Effect of human gonadortophins on spermiation and androgen biosynthesis in the testis of the toad *Bufo arenarum *(Amphibia, Anura) J Exp Zool 2006 305A 96 102 10.1002/jez.a.254 16358275

[B12] Mansour N Lahnsteiner F Patzner RA Collection of gametes from live axolotl, *Ambystoma mexicanum*, and standardization of *in vitro *fertilization Theriogenology 2010 in press 10.1016/j.theriogenology.2010.09.006 20965554

[B13] Trottier TM Armstrong JB Hormonal stimulation as an aid to artificial lnsemlnation in *Ambystoma mexicanum* Can Jf Zool 1975 53 171 173 10.1139/z75-021 1116071

[B14] Rowson AD Obringer AR Roth TL Non-invasive treatments of luteinizing hormone-releasing hormone for inducing spermeation in American (*Bufo americanus*) and Gulf coast (*Bufo valliceps*) toads Zoo Biol 2001 20 63 74 10.1002/zoo.1007 11429778

[B15] Sandow J The regulation of LHRH action at the pituitary and gonadal receptor level: a review Psychoneuroendocrinology 1983 8 277 297 10.1016/0306-4530(83)90003-3 6316392

[B16] Conn M The molecular basis of gonadotropin-releasing hormone action Endocr Rev 1986 7 3 10 10.1210/edrv-7-1-3 3007080

[B17] Browne R Li H Seratt J Kouba A Progesterone improves the number and quality of hormone induced Fowler toad (*Bufo fowleri*) oocytes Reprod Biol Endocrinol 2006 4 10.1186/1477-7827-4-3 PMC1373633 16451718

[B18] Trudeau VL Somoza GM Natale GS Pauli B Wignall J Jackman P Doe K Schueler FW Hormonal induction of spawning in 4 species of frogs by coinjection with gonadotropin-releasing hormone agonist and dopamine antagonist Reprod Biol Endocrinol 2010 8 36 10.1186/1477-7827-8-36 20398399 PMC2873446

[B19] Diakow C Raimondi D Frog reproductive behaviour BioScience 1981 31 53 56 10.2307/1308181

[B20] Boyd SK Arginine vasotocin facilitation of advertisement calling and call phonotaxis in Bullfrogs Horm Behav 1994 28 232 240 10.1006/hbeh.1994.1020 7814004

[B21] Toyoda F Yamamoto K Ito Y Tanaka S Yamashita M Kikuyama S Involvement of arginine vasotocin in reproductive events in the male newt *Cynops pyrrhogaster* Horm Behav 2003 44 346 353 10.1016/j.yhbeh.2003.06.001 14613729

[B22] Moore FL Zoeller RT Endocrine control of amphibian sexual behavior: evidence for a neurohormone-androgen interaction Horm Behav 1979 13 207 213 10.1016/0018-506X(79)90038-2 552369

[B23] Moore FL Wood RE Boyd SK Sex steriods and vasotocin interact in a female amphibian (*Taricha granulosa*) to elicit female-like egg-laying behaviour or male-like courtship Horm Behav 1992 26 156 166 10.1016/0018-506X(92)90039-X 1612562

[B24] Guillette LJ Jones RE Arginine vasotocin-induced in vitro oviductal contractions in *Anolis carolinensis*: effects of steroid hormone pretreatment in vivo The J Exp Zool 1980 212 147 152 10.1002/jez.1402120119

[B25] Guillette LJ Norris DO Norman MF Response of amphibian (*Ambystoma tigrinum*) oviduct to arginine vasotocin and acetylcholine *in vitro*: influence of steroid hormone pretreatment *in vivo* Comp Biochem Physiol 1985 8 151 154 10.1016/0742-8413(85)90147-1 2858337

[B26] Guillette LJ Jones RE Further observations on arginine vasotocin-induced oviposition and parturition in lizards J Herpetol 1982 16 140 144 10.2307/1563806

[B27] Zoeller RT Lais LT Moore FL Contractions of amphibian wolffian duct in response to acetylcholine, norepinephrine, and arginine vasotocin J Exp Zool 1983 226 53 57 10.1002/jez.1402260108 6304230

[B28] Watson GF Martin AA Life History, larval morphology and relationships of Australian Leptodactylid frogs Trans R Soc S Aust 1973 97 33 45

[B29] García-González F Simmons LW Sperm viability matters in insect sperm competition Curr Biol 2005 15 271 275 10.1016/j.cub.2005.01.032 15694313

[B30] Waggener WL Carroll EJ A method for hormonal induction of sperm release in anurans (eight species) and *in vitro *fertilization in *Lepidobatrachus *species Dev Growth Differ 1998 40 19 25 10.1046/j.1440-169X.1998.t01-5-00003.x 9563907

[B31] Sherman CDH Uller T Wapstra E Olsson M Within-population variation in ejaculate characteristics in a prolonged breeder, Peron's tree frog, *Litoria peronii* Naturwissenschaften 2008 95 1055 1061 10.1007/s00114-008-0423-7 18618091

[B32] Mylonas CC Fostier A Zanuy S Broodstock management and hormonal manipulations of fish reproduction Gen Comp Endocrinol 2010 165 516 534 10.1016/j.ygcen.2009.03.007 19318108

[B33] Rottmann RW Shireman JV Chapman FA Hormonal control of reproduction in fish for induced spawning Southern Regional Aquaculture Center 1991 Publication No. 424

[B34] Ciarcia G Angelini F Picariello O Botte V Arginine-vasotocin and gonadal activity in the lizard, *Podarcis s. sicula *Raf Experientia 1983 39 615 616 10.1007/BF01971125 6852199

[B35] Adashi EY Hsueh AJW Direct inhibition of testicular androgen biosynthesis revealing antigonadal activity of neurohypophysial hormones Nature 1981 293 650 652 10.1038/293650a0 7290200

[B36] Adashi EY Hsueh AJW Direct inhibition of rat testicular androgen biosynthesis by arginine vasotocin J Biol Chem 1982 257 1301 1308 6276379

[B37] Vaughan MK Vaughan GM Klein DC Arginine vasotocin: effects on development of reproductive organs Science 1974 186 938 939 10.1126/science.186.4167.938 4469691

[B38] Yamashita K Mieno M Yamashita ER Suppression of the luteinizing hormone releasing effect of luteinizing hormone releasing hormone by arginine-vasotocin J Endocrinol 1979 81 103 108 10.1677/joe.0.0810103 381560

